# Antimalarial Activity of Ethyl Acetate Extract and Fraction of *Bidens pilosa* against *Plasmodium berghei* (ANKA)

**DOI:** 10.1155/2020/8832724

**Published:** 2020-09-11

**Authors:** Noumedem Anangmo Christelle Nadia, Yamssi Cédric, Simeni Njonnou Sylvain Raoul, Ngongang Ouankou Christian, Mounvera Abdel Azizi, Gangueu Djape Clotilde Diane, Vanessa Rosine Nkouayep, Yondo Jeanette, Tsila Henri Gabriel, Mpoame Mbida

**Affiliations:** ^1^Department of Microbiology, Haematology and Immunology Faculty of Medicine and Pharmaceutical Sciences, University of Dschang, P.O. Box 96, Dschang, Cameroon; ^2^Department of Biomedical Sciences, Faculty of Health Sciences, University of Bamenda, P.O. Box 39, Bambili, Cameroon; ^3^Department of Internal Medicine and Specialties, Faculty of Medicine and Pharmaceutical Sciences, University of Dschang, P.O. Box 96, Dschang, Cameroon; ^4^Department of Animal Biology, Faculty of Science, University of Dschang, P.O. Box 067, Dschang, Cameroon

## Abstract

**Background:**

Malaria is one of the most critical diseases causing about 219 million cases worldwide in developing countries. The spread and development of resistance against chemical antimalarial drugs is one of the major problems associated with malaria control. The present study was to investigate the antimalarial efficacy of ethyl acetate extract and one fraction of *Bidens pilosa in vivo* in order to support the usage of this plant by traditional healers to treat malaria.

**Methods:**

The extracts were prepared by maceration of *B. pilosa* leaf powder in ethyl acetate. The liquid filtrate of the extract and the best *in vitro* antiplasmodial fraction using HPLC were concentrated and evaporated using a rotavapor under vacuum to dryness. The antimalarial activity of *B. pilosa* plant products were evaluated *in vivo* against *Plasmodium berghei* infected mice according to the Peter and Rane test. The antimalarial efficacy of the a selected crude extract (ethyl acetate extract) was evaluated at 125, 250, and 500 mg/kg, while a selected fraction from ethyl acetate extract (fraction 12) was evaluated at 62.5 and 125 mg/kg. Blood from experimental animals was collected to assess hematological parameters.

**Results:**

The crude extract of ethyl acetate and fraction 12 demonstrated 100% *in vivo* parasite suppressive activity at doses of 500 mg/kg and 125 mg/kg, respectively, for the crude extract and fraction 12. The mice treated with 250 and 500 mg/kg had their parasitemia (intraerythrocytic phase of *P. Berghei*) drop considerably, disappearing by the 8^th^ day in mice receiving 500 mg/kg. The ethyl acetate extract of *B. pilosa*, fraction 12 showed an even higher antiplasmodial activity. By the 5^th^ day of the experiment, the treatment led to a modification of hematological parameters in mice. The chloroquine (5 mg/kg), fraction 12 (125 mg/kg), and the crude extract (500 mg/kg) groups all survived the 30 days of the experiment, while the negative control group registered 100% of the deaths.

**Conclusion:**

This study scientifically supports the use of *Bidens pilosa* leaves in the traditional treatment of malaria. However, the mode of action and *in vivo* toxicity of the plant still need to be assessed.

## 1. Background

Since ancient times, malaria has posed and is still posing a substantial public health problem to many African countries. Epidemiological studies and data from national health surveys show that malaria is still the leading cause of child morbidity and mortality in Africa. According to the WHO estimates, approximately 219 million people suffered from malaria in 2017 [1].

In many African countries, the causing parasites have developed resistance to the “old” affordable drug chloroquine and to the sulphadoxine pyrimethamine [2]. Therefore, artemisinin combination therapy has been adopted as a first line of treatment for uncomplicated malaria. However, for operational and cost reasons, these drugs are not readily available. The search for new drugs is therefore an important challenge [3].

In Africa, more than 80% of the population relies on traditional medicine based on plant preparations to cure or prevent malaria [4]. Therefore, the WHO encourages endemic countries to evaluate local antimalarial remedies for their efficacy and safety and to support initiatives for the development of standardized, quality-controlled preparations and products [4]. *Bidens pilosa* is a medicinal plant which is used by traditional healers in Cameroon to treat certain diseases such as helminthiasis, typhoid, and malaria [5]. More to that, many previous works [6, 7] have demonstrated the antimalarial efficacy of the various extracts of *B. pilosa*. Brandão et al. [8] demonstrated that the ethanolic extract of *Bidens pilosa* contains flavonoids and acetylenes which are responsible for its antiplasmodial activity. Noumedem Anangmo et al. [5] demonstrated the antiplasmodial efficacy of *Bidens pilosa* on three strains of *P. falciparum.* According to Andrade-Neto et al. [6], *Bidens pilosa* has antimalarial efficacy at doses less than 500 mg/kg.

In fact, *Bidens pilosa* ethyl acetate extract and its most effective fraction (fraction 12) were reported to be highly active against *P. falciparum in vitro* and noncytotoxic against L929 cells using MTT assay [5].

*In vitro* tests assume direct action on parasites and are not sufficient to attest antimalarial efficacy. Results obtained under *in vitro* conditions cannot be extrapolated *in vivo* taking into consideration biotransformation, interaction with food, enzymatic material, and absorption observed *in vivo* [9]. Therefore, the antimalarial activity of the extract must be verified *in vivo*. The aim of this study is to assess the antimalarial efficacy of ethyl acetate extract and fraction 12 of *Bidens pilosa in vivo* to support traditional healers' usage of the plant for malaria treatment.

## 2. Material and Methods

### 2.1. Plant Material

In March 2015, fresh *Bidens pilosa* leaves were collected from Dschang-Cameroon. Mr. Victor Nana, a plant taxonomist, identified the plant and deposited a voucher specimen at Cameroon's National Herbarium (Yaounde) under accession number 18572/SRF-CAM.

### 2.2. Preparation of Extract and Fraction 12

Extract of ethyl acetate was obtained using the method defined by Wabo Pone et al. [10]. The extract was prepared by maceration using ethyl acetate solvent. Briefly, 100 g of stored powder was kept in ethyl acetate for 72 hours. The liquid filtrate of the extract was concentrated and evaporated using a rotavapor (BUCHI R-210) under vacuum (40°C) to dryness. The ethyl acetate extract was introduced into a semiprep reverse-phase HPLC chromatogram to obtain the various fractions, and fraction 12 presented the best *in vitro* antiplasmodial activity [5]. The extract and fraction 12 were stored in a refrigerator at 4°C, for further *in vivo* antiplasmodial processing.

### 2.3. *In Vivo* Pharmacological Studies

#### 2.3.1. Preparation of the Parasite Inoculum

The inoculum consisted of 5 × 10^7^*P. berghei* (ANKA) erythrocytes per milliliter. This was obtained from a donor mouse.

#### 2.3.2. The 4-Day Suppressive Test

The antimalarial activity of the crude extract and fraction 12 were determined using Peter's 4-day suppressive test [11]. Forty-eight (48) Swiss albino mice (male and female) of average weight 22 g were distributed into 9 groups of 6 mice each as follows:

1. Group 1: Infected and treated with crude extract at 500 mg/kg body weight.
2. Group 2: Infected and treated with crude extract at 250 mg/kg body weight.
3. Group 3: Infected and treated with crude extract at 125 mg/kg body weight.
4. Group 4: Infected and treated with fraction 12 at 125 mg/kg body weight.
5. Group 5: Infected and treated with fraction 12 at 62.5 mg/kg body weight.
6. Group 6 Infected and treated with chloroquine at 5 mg/kg body weight (positive control).
7. Group 7: Infected and treated with 10% hypromellose (negative control).
8. Group 8: Noninfected–nontreated (neutral control).

The first seven (7) groups were infected intravenously on day zero with 1 × 10^7^ of parasitized red blood cells. Three hours later, the crude extract, fraction 12, and chloroquine were administered to the animals orally. These mice were treated every 24 hours for 4 days (day 0, day 1, day 2, and day 3). Thin blood films were prepared from the tail of each animal on the 4^th^ day till 9^th^ to determine the parasitemia and percentage of inhibition. The average suppression of parasitemia was calculated as follows [11]:

(1) $$\begin{array}{c} \text{Average percentage supression}=\frac{\%\text{Parasitemia control}-\%\text{parasitemia treatment group}}{\%\text{parasitemia control}} \end{array}$$

The mice's mean survival time in each treatment group was determined over a 29-day (D0-D28) period, as follows [11]: (No.of days survived)/(total No.of days (30) × 100).

#### 2.3.3. Curative Activities of Ethyl Acetate Extract and Fraction 12 of *B. pilosa*

The curative test was conducted according to Ryley and Peters [12].

For this, 30 male and female mice infected intravenously on day 0 with 1 × 10^7^ parasitized erythrocytes were divided into 5 groups of 6 mice. Seventy-two hours (72 h) after the infection, the experimental animals were treated on the 4^th^ day, 5^th^ day, 6^th^ day, and 7^th^ day.

1. Group 1: Infected and treated with crude extract at 500 mg/kg body weight.
2. Group 2: Infected and treated with fraction 12 at 125 mg/kg body weight.
3. Group 3: Infected and treated with chloroquine at 5 mg/kg body weight (positive control).
4. Group 4: Infected and treated with 10% hypromellose (negative control).
5. Group 5: Noninfected–nontreated (neutral control).

From the 4^th^ day till 8^th^ day, parasitemia in all the experimental groups was determined by the May-Grünwald-Giemsa staining technique. The average survival rate of the different treatment groups was determined arithmetically by calculating the average survival time in days from the day of infection to the 30^th^ day (day 0-day 29). The activity of the products on the parasites was expressed as a function of the reduction in the parasitemia of the treated mice compared to the mice of the control group [13]. The mean reduction rate of parasitemia was calculated as: %reduction = 100 × [(C–T)/C], where *C* is the average parasitemia in the control group and *T* the average parasitemia in the treated group [12].

### 2.4. Blood Sampling for Hematological Analyses

The addition of three mice from each treatment group on the 6th day after treatment was anaesthesized under chloroform and sacrificed, and about 1 ml of blood obtained from each mouse through cardiac puncture into tubes already coated with EDTA was used for hematological analysis using automated hematology analyser as describe by Asanga et al. [14].

### 2.5. Statistical Analysis

Results were expressed as mean ± standard deviation. The effects of the extract and fraction were evaluated using one way ANOVA (Analysis of variance), followed by the Duncan test for means separation when a significant difference existed. The Chi^2^ test was performed to compare the mortality rate. For all analyses, the limit of significance was 5%, and the SPSS 20.0 software was used.

## 3. Results

### 3.1. Suppressive Effect of Crude Extract and Fraction 12 on the Development of *Plasmodium berghei* Parasite

**Figure 1:**
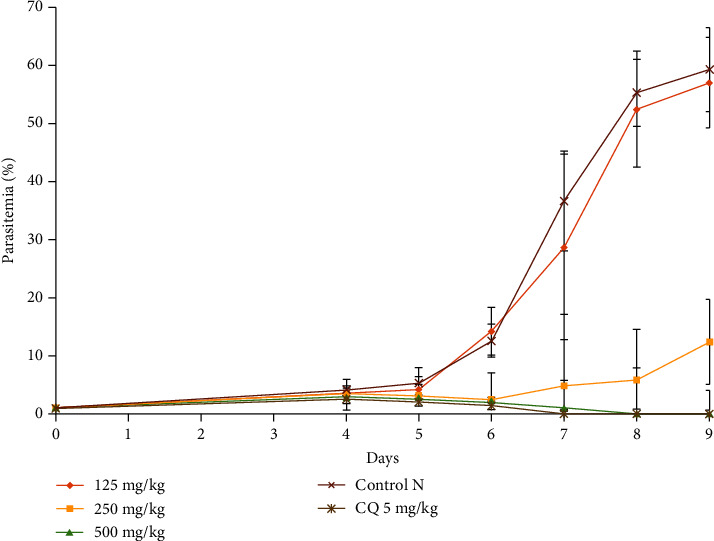
Effect of ethyl acetate extract of *B. pilosa* on the evolution of *Plasmodium berghei* parasitemia with respect to time.

Figure 1 shows the evolution of *P. berghei* parasitemia in infected mice treated with different doses of the crude extract *B. pilosa.* It appears that the dose of 125 mg/kg does not influence the development of the erythrocytic phase of *P. berghei*, which results to an increase in parasitemia between days 5 and 9. Whereas, mice treated with doses of 250 and 500 mg/kg had their parasitemia drop considerably and disappear by the 8^th^ day in mice receiving the dose 500 mg/kg.

**Figure 2:**
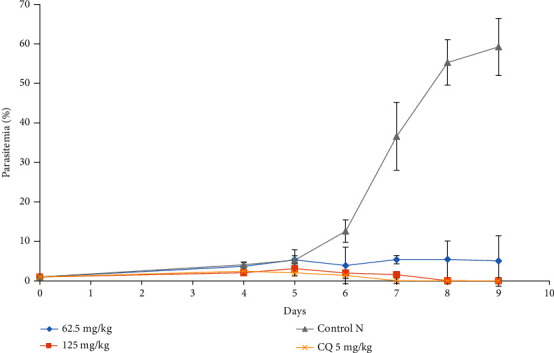
Effect of fraction 12 of the ethyl acetate extract of *B. pilosa* on the evolution of *Plasmodium berghei* parasitemia in mice with respect to time.

Fraction 12 from the *B. pilosa* ethyl acetate extract also showed antiplasmodial activity (Figure 2). However, this activity was more important than that of the crude extract. It appears from this figure that the doses of 62.5 and 125 mg/kg had a parasitemia lower than 5%. This rate was 0% by the 8^th^ day of the experiment at the dose of 125 mg/kg and % for chloroquine. The animals that received no treatment had their parasitemia reaching 60% on the 9^th^ day of the experiment.

### 3.2. Determination of Mean Survival Time

From the time of infection until death, mortality of each mouse was monitored and recorded regardless of the group in which the mouse was allocated throughout the follow-up period (30 days). Mean survival time of mice of each group was determined using the following formula:

Table 1 shows the suppressive effect, parasitemia level, and mean survival rate of crude extract and fraction 12 on the development of *Plasmodium berghei* after the experiment. It appears that all the animals that received chloroquine crude extract (500 mg/kg) and fraction 12 (125 mg/kg) survived 30 days posttreatment. On the other hand, 100% deaths were recorded in the negative control group and those treated with the crude extract at a dose of 125 mg/kg.

### 3.3. Effect of Crude Extract and Fraction 12 on Hematological Parameters

Table 2 presents the effect of the crude extract and fraction 12 of *Bidens pilosa* on hematological parameters. The 5^th^ day of treatment led to a modification of hematological parameters. For example, in the negative control group, there was an increase in white blood cell count, mean platelet volume, and the RBC distribution index of red blood cells. All the other parameters decreased compared to the neutral control group. In the group treated with the crude extract, fraction 12, and chloroquine, parameters such as hemoglobin, red blood cells, and hematocrit increased significantly (*P* < 0.05) compared to the negative control group and lower than the neutral control group. Other parameters, such as the mean corpuscular volume, mean corpuscular hemoglobin content, and mean corpuscular hemoglobin concentration, were slightly modified without significant difference (*P* > 0.05) as compared to the neutral and negative control groups.

### 3.4. Curative Effect of the Crude Extract and Fraction 12 of Ethyl Acetate on the Development of *Plasmodium berghei*

**Figure 3:**
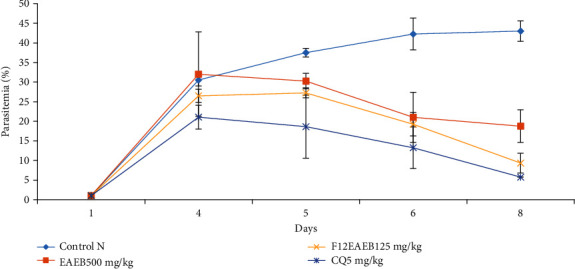
Curative effect of the crude extract and fraction 12 of *B. pilosa* on the evolution of *Plasmodium berghei* parasitemia in mice with respect to time.

Figure 3 shows the evolution of the parasitemia of *P. berghei* in infected mice and treated 3 days later with 500 mg/kg of crude extract and 125 mg/kg of fraction 12. It may be seen that all the products administered produced a reduction in the parasitemia of *P. berghei* after repeated administration of these products.

### 3.5. Determination of Mean Survival Time after Treatment with Crude Extract and Fraction 12

Table 3 shows the reductive effect, the parasitemia level, and the mean survival rate of crude extract and fraction 12 on the development of *Plasmodium berghei* after the curative test. It appears that all the animals in the chloroquine (5 mg/kg), fraction 12 (125 mg/kg), and the crude extract (500 mg/kg) groups survived the 30 days of the experiment. However, 100% of the deaths were recorded in the negative control group.

## 4. Discussion

The antiplasmodial activity of many plants of the Asteraceae family has already been demonstrated [15]. This would be due to the presence of classes of secondary metabolites which would act at the level of the digestive vacuole of the parasite, because the two main classes of antimalarial drugs (quinolines and artemisinin derivatives) of molecules contained in plants of the family of Asteraceae would act on the parasite's digestive vacuole [16]. Moreover, Noumedem Anangmo et al. [5] demonstrated the antiplasmodial efficacy of *Bidens pilosa* on three strains of *Plasmodium falciparum.*

The suppressive test is commonly used as a standard antimalarial efficacy test. The effectivity of the *P. berghei* artificial infection is attested by the exponential increase in parasitemia in mice by the 4^th^ day postinfection [13].

The level of parasitemia was significantly lower in the treated compared to the untreated group. The reference drug used in this study (chloroquine 5 mg/kg of body mass) exerted a 100% suppression which is expected according to Kiseko et al. [17]. *Plasmodium berghei* growth was completely inhibited in mice by the ethyl acetate extract (500 mg/kg) and fraction 12 (125 mg/kg). Similarly, Andrade-Neto et al. [6] reported that the ethanol extracts of *B. pilosa* were effective in mice at 500 mg/kg. The better efficient observe with the fraction 12 can be due to the fact that this fraction contained mainly the antiplasmodial metabolites. Meanwhile, the constituents of crude extract are diluted by other metabolites. The reduction in parasitemia was even more significant from the fourth day. This may be as a result of an accumulation of secondary metabolites in blood following the administration of repeated doses of the tested products which penetrate the parasites, exerting their effect [18].

Biochemical and hematological indices are reliable parameters for assessing the health status of animals [19]. White blood cells play a role in the immune defense against foreign bodies, generally through leukocytosis and the production of antibodies [20]. The number of white blood cells in the negative control group increases significantly (*P* > 0.005) compared with the treated groups. Odeghe Othuke et al. [21] found a contrary trend in *P. berghei*-infected mice and treated with *Anthocleista grandiflora* methanolic extract. The lower white blood cell count in infected mice may be due to reduced resistance of mice to infection [22]. Leukocytosis in the negative control group may be due either to a bone marrow tumor, leukemia, or tissue breakdown and inflammatory disease in mice infected with *P. berghei* [23]. We found a substantial increase (*P* < 0.05) in the concentration of hemoglobin, hematocrit, and red blood cells in the chloroquine-treated groups and crude extract of *B. pilosa* relative to the negative control mice. The extract may have inhibited the development of parasites and therefore preventing a drastic reduction in the concentration of hemoglobin, hematocrit, and red blood cells, which are indicators of severe anaemia [24]. The anaemia observed in infected and untreated animals is likely due to the destruction of red blood cells, caused by the multiplication of the parasite or by the activity of reticuloendothelial cells in the spleen [22]. This decrease in red blood cells may also be as a result of several other factors, among which is the repeated hemolysis of infected erythrocytes [24]. One of the parameters used to define the antimalarial efficacy of a tested product is the average survival rate. By the 9^th^ day of the experiment, we recorded 100% mortality in the negative control group. However, no mice died in the groups treated with chloroquine at a dose of 5 mg/kg of body weight. A similar observation was made by Okokon et al. [25] when evaluating the antimalarial activity of cornsilk extracts and fractions of *Zea Mays* on *P. berghei.* The total survival of animals in the positive control demonstrates the sensitivity of the *Plasmodium* strain to chloroquine [11]. All animals treated with 500 mg/kg of crude extract and 125 mg/kg of fraction 12 survived at the end of the experiment. Ajala et al. [26] obtained the same result when studying the effect of extracts of *Phyllanthus amarus* on *Plasmodium yoelii.* The survival of the treated animals may be due to the bioactive compounds (antimalarial) contained in the natural substances tested.

As in the suppressive test, the curative effect of the crude extract (500 mg/kg) and fraction 12 (125 mg/kg) was equally determined, and fraction 12 showed a greater curative antimalarial activity. However, the curative effect of the products tested remained lower than their suppressive effect. At doses of 500 mg/kg for ethyl acetate extract and 125 mg/kg for fraction 12, a 100% suppression was obtained. However, at these same doses, the curative effect of these extracts was less than 80%. Similar results were obtained by Dhanabalan et al. [27]. When testing the *in vivo* antiplasmodial activity of four Indian medicinal plants, these authors concluded that the parasites were more sensitive to the curative test than to the suppressive test [27]. According to Noumedem et al. [5], a few molecules present within the plant extract may be related to the recorded antimalarial activities of *B. pilosa*, particularly the Phenol, 2, 4-Bis (1, 1-Dimethylethyl), a known antiplasmodial molecule. The Phenol, 2, 4-Bis (1, 1-Dimethylethyl) had 0.71% in the crude extract and 15.20% in fraction 12. Clearly, *B. pilosa* shows definite antimalarial activity which justifies its use in the treatment of malaria in West Cameroon.

## 5. Conclusion

There is an urgent need for the discovery and development of new chemotherapeutic compounds to meet with the challenges of drug resistance. The ethyl acetate extract and fraction 12 of *B. pilosa* contain antiplasmodial compounds. This study scientifically gives support to the use of *B. pilosa* to treat malaria in folkloric medicine. However, toxicological and pharmacological studies are required to move forward in the exploitation of *B. pilosa* as a new antimalarial agent.

## Figures and Tables

**Table 1:** Suppressive effect, parasitemia level, and mean survival rate of crude extract and fraction 12 on the development of *Plasmodium berghei*.

Group	Level of parasitemia	Percent suppression	Mean survival date
Crude extract			
125 mg/kg	56.98 ± 1, 4	3.81 ± 0.08	11 ± 0.8
250 mg/kg	12.32 ± 1.3	79.20 ± 1.18	22.5 ± 0.52
500 mg/kg	0 ± 0	100 ± 0	30 ± 0
CQ 5 mg/kg	0 ± 0	100 ± 0	30 ± 0
Fraction 12			
62,5 mg/kg	5.05 ± 0.81	91.48 ± 1.42	27.5 ± 1.5
125 mg/kg	0 ± 0	100 ± 0	30 ± 0

**Table 2:** Effect of the crude extract and fraction 12 on hematological parameters.

PH treatments	Doses (mg/kg)	WBC (×10^9^/l)	RBC (×10^12^/l)	PL (%)	MPV (fl)	PDW	HGB (g/dl)	HCT (%)	MCV (fl)	MCHC (Pg)	MCHC (g/dl)	DIRBC (%)
Neutral control	0	7.6 ± 1.1^∗^	9.10 ± 0.5^∗^	0.45 ± 0.05	6.60 ± 0.20	14.20 ± 0.10	14.3 ± 1.60^∗^	48.3 ± 0.8^∗^	54.22 ± 3.40	15.70 ± 0.70	31.10 ± 0.20	17.70 ± 0.50
Negative control		14.10 ± 2.10	6.80 ± 0.40	0.32 ± 0.07	6.90 ± 0.20	14.50 ± 0.20	8.70 ± 1.20	32.2 ± 6.50	53.30 ± 3.20	14.80 ± 0.40	29.10 ± 1.20	18.10 ± 0.10

CQ	5	9.10 ± 0.60^∗^	8.20 ± 0.43^∗^	0.39 ± 0.06	6.70 ± 0.10	14.10 ± 0.20	10.60 ± 1.20^∗^	44.10 ± 0.40^∗^	54.10 ± 2.40	15.00 ± 0.40	31.40 ± 0.30	17.90 ± 0.20

Crude extract	**125**	10.5 ± 1.03^∗^	8.30 ± 0.23^∗^	0.37 ± 0.03	7.00 ± 0.10	14.3 ± 0.20	12.10 ± 0.20^∗^	41.2 ± 0.9^∗^	52.11 ± 0.50	14.70 ± 0.60	29.00 ± 0.20	17.70 ± 0.41
	**250**	11.4 ± 0.20^∗^	8.68 ± 0.22^∗^	0.50 ± 0.13	6.90 ± 0.20	14.90 ± 0.30	11.60 ± 0.3^∗^	40.11 ± 2.1^∗^	52.00 ± 0.30	13.90 ± 0.90	27.23 ± 0.37	17.60 ± 0.40
	**500**	10.40 ± 0.23^∗^	8.70 ± 0.40^∗^	0.31 ± 0.05	7.00 ± 0.20	14.60 ± 0.20	13.40 ± 0.40^∗^	42.7 ± 5.40^∗^	52.20 ± 0.70	14.90 ± 0.50	28.80 ± 0.23	17.20 ± 0.22

Fraction 12	**62.5**	11.12 ± 0.41^∗^	8.05 ± 0.06^∗^	0.39 ± 0.32	6.96 ± 0.06	14.18 ± 0.32	9.36 ± 0.15^∗^	42.61 ± 0.31^∗^	53.89 ± 0.12	13.64 ± 0.62	29.06 ± 0.08	17.83 ± 0.14
	125	10.32 ± 0.11^∗^	8.21 ± 0.07^∗^	0.43 ± 0.28	6.53 ± 0.02	14.13 ± 0.14	9.86 ± 0.31^∗^	43.13 ± 0.75^∗^	54.17 ± 0.58	14.93 ± 0.13	30.02 ± 0.31	17.94 ± 0.74

HCT: hematocrit; HGB: hemoglobin; MCHC: mean corpuscular hemoglobin concentration; MCV: mean corpuscular volume; PL: platelets; DIRBC: distribution index of red blood cells; MPV: mean platelet volume.

**Table 3:** Parasitemia level, reductive effect, and mean survival time of crude extract and fraction 12 on the development of *Plasmodium berghei* after the curative test.

Treatments	Dosage (mg/kg)	Level of parasitemia	Percentage supression	Mean survival time
Hypromellose 10%	0	43.01 ± 1.58	0	/
Chloroquine	5	5.75 ± 0.40	86.63 ± 1.32	30 ± 0
Crude extract	500	18.75 ± 1.23	56.41 ± 1.15	24.17 ± 0.41
Fraction 12	125	9.33 ± 0.03	78.31 ± 0.91	30 ± 0

## Data Availability

Data and material are available to other researchers upon request.
